# Elimination of SARS-CoV-2 in nasopharynx and oropharynx after use of an adjuvant gargling and rinsing protocol with an antiseptic mouthwash

**DOI:** 10.31744/einstein_journal/2021CE6999

**Published:** 2021-12-20

**Authors:** Fabiano Vieira Vilhena, Bernardo da Fonseca Orcina, Lúcio Lemos, Jeanette Cecília Fournier Less, Isabella Pinto, Paulo Sérgio da Silva Santos

**Affiliations:** 1 TRIALS Bauru SP Brazil Trials - Saúde Bucal & Tecnologias - TRIALS, Bauru, SP, Brazil.; 2 Faculdade de Odontologia de Bauru Universidade de São Paulo Bauru SP Brazil Faculdade de Odontologia de Bauru, Universidade de São Paulo, Bauru, SP, Brazil.; 3 Lemos Laboratórios de Análises Clínicas Juiz de Fora MG Brazil Lemos Laboratórios de Análises Clínicas, Juiz de Fora, MG, Brazil.

Dear Editor,

It has been well established in science that the mouth is an entry and site of replication of the severe acute respiratory syndrome coronavirus 2 (SARS-CoV-2), which causes the coronavirus 2019 disease (COVID-19). Hence, oral health care and use of broad-spectrum antimicrobial products become relevant.^(1)^ Recently the use of phthalocyanine derivative mouthwash (PDM), has been investigated, demonstrating *in vitro* activity in reduction of viral load of SARS-CoV-2, in addition to clinical improvement and less severe disease, with no reports of adverse effects in COVID-19 patients.^(1,2)^The present study addressed the effect of PDM use in reducing the viral load of the novel coronavirus in a series of cases comprising four adult patients, who work together and tested positive for SARS-CoV-2 between June 16 and 21, 2021. The ethical precepts were complied with, and the study was approved by the Human Research Ethics Committee (opinion number: 4,273,068, CAAE: 36493520.1.0000.5417). All patients presented negative routine tests in the previous week. Diagnosis of SARS-CoV-2 and monitoring of viral load were made by real-time polymerase chain reaction with reverse transcriptase (RT-PCR), using fluorescent probes (Taqman) (Thermo Fischer Scientific). Nucleic acids were extracted from 100μL of nasopharyngeal/oropharyngeal swab, using the virus RNA + DNA Preparation Kit, as per the manufacturer´s instructions (Cellco, São Carlos, SP, Brazil). The purified nucleic acid was reversely transcript into cDNA, and amplified using the kit TaqPath™ COVID-19 CE IVD RT PCR (European Commission - *in vivo* Diagnostics Real Time - Polymerase Chain Reaction) (Applied Biosystems^TM^), in equipment 7500 Fast Real-Time PCR Systems (Applied Biosystems^TM^). The technique employed was the cycle threshold (CT) of each sample, which corresponds to the number of cycles of PCR required to identify the virus. The kit used assessed markers for SARS-CoV-2 with sensibility greater than 99%, and specificity of 100%, with detection limit of 2 copies/mL. The RT-PCR was considered positive when two or more genes assessed (ORF1ab, N and S) presented CT <37. The mutations on gene S of the virus were also investigated, and could be one of the following originating from the original strains: P1 (Manaus), P2 (Brazil), B1.1.7 (British) and B1.351 (South African) (CDC-EUA).^(3)^

After diagnosis, the clinical treatment prescribed to patients comprised use of analgesics, antiflu agents, vitamins C and D, in addition to an adjuvant protocol of gargling/rinsing with PDM (5mL), for 1 minute, five times a day, for seven days.

As shown in table 1, the participants had no underlying medical conditions, except patient D - hypertension controlled with medicine, and were asymptomatic, or with mild flu symptoms, one day before the positive test. Most participants reported history of mild COVID-19, and case A stood out, stating testing positive for SARS-CoV-2 during 22 consecutive days, in the first infection. All patients were diagnosed as strain P1 of SARS-CoV-2, with viral load ranging from 176 to 111,750 copies/mL. Up to 72 hours after the first positive test/starting on PDM protocol, 100% of patients tested negative for a second RT-PCR test of swab to viral load of SARS-CoV-2 in nasopharynx and oropharynx, and were asymptomatic.

**Table 1:** Clinical and laboratory data of patients in the case series

Patient	Sex	Age	Pre-test symptoms	Comorbidity/history of COVID-19	Days of symptoms before of the test	Initial PCR (Mean CT)	Initial number of copies/mL	Strain SARS-CoV-2	Interval between initial PCR and negative PCR tests
A	M	58	Choriza	No comorbity / March 2020 and February 2021	1	30.5	1,113	P1	24 hours
B	F	53	Asymptomatic	No comorbity / February 2021	-	17.6	111,750	P1	72 hours
C	F	45	Asymptomatic	No comorbity / September 2020 and February 2021	-	33.5	176	P1	72 hours
D	F	29	Choriza, headache and malaise	Hypertension / no history of COVID-19	1	26.6	1,898	P1	72 hours

CT: cycle threshold; SARS-CoV-2: acute respiratory syndrome coronavirus 2; PCR: polymerase chain reaction.

These results are in agreement with the current literature, since there is scientific evidence on the reduction of viral load of SARS-CoV-2 by some mouthwash.^(1,4)^ Moreover, the use of oral hygiene products able to inactivate SARS-CoV-2 have been recommended and used as additional preventive measure against COVID-19.^(1,2)^ SARS-CoV-2 variants have emerged, including gamma variant (P1), known as the most lethal and of high transmissibility, due to mutation in gene S.^(5,6)^ There are reports of reinfections that could be associated to different strains of SARS-CoV-2, and of patients with mild COVID-19, who may have controlled replication of SARS-CoV-2 and not developed detectable humoral immunity. In this case series of positive SARS-CoV-2 variant P1, most reported history of mild COVID-19 before the present diagnosis. Asymptomatic individuals can disseminate the virus, which demonstrates the importance of correct epidemiological measures to identify, isolate and inactivate the contaminants. The sample size and the lack of adjuvant control therapy could be mentioned as limiting factors in this case series. Nonetheless, the quick elimination of the virus in the nasopharynx and oropharynx of individuals included in the PDM gargling/rinsing protocol is a promising result in controlling SARS-CoV-2 spreading, and should be object of future research.
