# Supplementary material for: Elimination of SARS-CoV-2 in nasopharynx and oropharynx after use of an adjuvant gargling and rinsing protocol with an antiseptic mouthwash
Source: Einstein (Sao Paulo). 2021 Dec 20;19:eCE6999. doi: 10.31744/einstein_journal/2021CE6999 (PMC8693883; doi:10.31744/einstein_journal/2021CE6999)
Supplement: Translation

# Translation: Eliminação de SARS-CoV-2 em nasofaringe e orofaringe após o uso de um protocolo adjuvante de gargarejo e bochecho com antisséptico bucal

**Authors:** Fabiano Vieira Vilhena, Bernardo da Fonseca Orcina, Lúcio Lemos, Jeanette Cecília Fournier Less, Isabella Pinto, Paulo Sérgio da Silva Santos

**Affiliations:** 1 Brasil Trials - Saúde Bucal & Tecnologias - TRIALS, Bauru, SP, Brasil.; 2 Brasil Faculdade de Odontologia de Bauru, Universidade de São Paulo, Bauru, SP, Brasil.; 3 Brasil Lemos Laboratórios de Análises Clínicas, Juiz de Fora, MG, Brasil.

Caro Editor,

É bem estabelecido na ciência que a boca é uma porta de entrada e sítio de replicação do coronavírus da síndrome respiratória aguda grave (SARS-CoV-2), que causa a doença do coronavírus 2019 (COVID-19), tornando relevante a atenção aos cuidados com a saúde oral e a utilização de produtos de amplo espectro antimicrobiano.^(1)^ Recentemente o uso de um antisséptico bucal contendo derivado de ftalocianina (ABF), vem sendo pesquisado, demonstrando atividade *in vitro* na redução da carga viral do SARS-CoV-2, bem como melhora clínica e menor gravidade da doença, sem relatos de efeitos adversos, em pacientes com COVID-19.^(1,2)^ O presente estudo abordou o efeito do uso do ABF na redução da carga viral do novo coronavírus em uma série de casos formada por quatro pacientes adultos, colegas de trabalho, diagnosticados como positivos para a presença de SARS-CoV-2 entre os dias 16 e 21 de junho de 2021. O estudo seguiu os preceitos éticos e foi aprovado pelo Comitê de Ética e Pesquisa em Seres Humanos (número do parecer: 4.273.068, CAAE: 36493520.1.0000.5417). Todos apresentaram exames de rotina negativos na semana anterior. O diagnóstico do SARS-CoV-2 e o monitoramento da carga viral foram realizados por reação em cadeia da polimerase em tempo real com transcriptase reversa (RT-PCR), utilizando sondas fluorescentes (Taqman) (*Thermo Fischer Scientific*). Os ácidos nucléicos foram extraídos a partir de 100μL de *swab* nasofaríngeo/orofaríngeo utilizando o vírus RNA + DNA Preparation Kit, seguindo as instruções do fabricante (Cellco, São Carlos, SP, Brasil). O ácido nucleico purificado foi transcrito reversamente em cDNA e amplificado usando o kit TaqPath™ COVID-19 CE- IVD RT - PCR (Comissão Européia - *in vivo Diagnostics Real Time - Polymerase Chain Reaction*) (Applied Biosystems^TM^) no equipamento 7500 *Fast Real-Time PCR Systems* (Applied Biosystems^TM^). A técnica utilizada foi o limite de ciclo (CT - *ciclo threshold*) de cada amostra, o que corresponde ao número de ciclos de PCR necessários para identificação do vírus. O kit utilizado avaliou marcadores para SARS-CoV-2 com sensibilidade maior que 99% e especificidade de 100% com limite de detecção de 2 cópias/mL. O RT-PCR foi considerado positivo quando dois ou mais genes avaliados (ORF1ab, N e S) apresentaram CT <37. Também foram investigadas as mutações localizadas no gene S do vírus, podendo ser uma das seguintes derivadas das linhagens originais: P1 (Manaus), P2 (Brasil), B1.1.7 (Britânica) e B1.351 (Sul Africana) (CDC-USA).^(3)^

A partir do diagnóstico, o tratamento médico estabelecido aos pacientes envolveu o uso de analgésico, antigripais, vitaminas C e D, além do uso do protocolo adjuvante de gargarejo/bochecho com ABF (5mL) durante 1 minuto/cinco vezes ao dia por sete dias.

Conforme tabela 1, os participantes não apresentavam comorbidades, exceto paciente D - hipertensão controlada por medicamento, e se encontravam assintomáticos ou com sintomas gripais leves um dia antes do exame positivo. A maioria dos participantes relatou histórico de COVID-19 leve, com destaque para o caso A que informou testar positivo por 22 dias consecutivos para o SARS-CoV-2 na primeira vez que se contaminou. Todos os pacientes foram diagnosticados com a cepa P1 do SARS-CoV-2, com carga viral que variou de 176 a 111.750 cópias/mL. Em até 72 horas após o primeiro exame positivo / início do protocolo ABF, 100% dos pacientes testaram negativo para um segundo exame de swab para RT-PCR de carga viral do SARS-CoV-2 em nasofaringe e orofaringe, e estavam assintomáticos.

**Tabela 1:** Dados clínicos e laboratoriais dos pacientes da série de casos

Paciente	Sexo	Idade	Sintomas pré exame	Comorbidade/ histórico de COVID-19	Dias de sintoma antes do exame	PCR inicial (CT médio)	Número de cópias/ml inicial	Cepa SARS-CoV-2	Intervalo entre os exames de PCR inicial e PCR negativo
A	M	58	Coriza nasal	Sem comorbidade/ março 2020 e fevereiro 2021	1	30,5	1.113	P1	24 horas
B	F	53	Assintomático	Sem comorbidade/ fevereiro 2021	-	17,6	111.750	P1	72 horas
C	F	45	Assintomático	Sem comorbidade/ setembro 2020 e fevereiro 2021	-	33,5	176	P1	72 horas
D	F	29	Coriza nasal, dor de cabeça e no corpo	Hipertensão/ sem histórico de COVID-19	1	26,6	1.898	P1	72 horas

CT: *cycle threshold*; SARS-CoV-2: *acute respiratory syndrome coronavirus* 2; PCR: *polymerase chain reaction*.

Estes resultados estão em consonância com a literatura atual, uma vez que existe evidência científica sobre a atividade de redução de carga viral do SARS-CoV-2 para alguns antissépticos orais.^(1,4)^ Além disso, o uso de produtos de higiene oral capazes de inativar o SARS-CoV-2 têm sido recomendados e utilizado como medida preventiva adicional contra a COVID-19.^(1,2)^ Variantes do SARS-CoV-2 tem surgido, dentre elas a variante gama (P1), conhecida como mais letal e de maior transmissibilidade devido a sua mutação no gene S.^(5,6)^ Há relatos de episódios de reinfecções que podem estar associadas a diferentes cepas de SARS-CoV-2, ou ainda de que pacientes com COVID-19 de grau leve podem ter controlado a replicação do SARS-CoV-2 sem desenvolver imunidade humoral detectável. Nesta série de casos de SARS-CoV-2 variante P1 positivos, a maioria relatou histórico de COVID-19 leve anteriormente ao diagnóstico atual. Indivíduos assintomáticos podem propagar o vírus, o que demonstra a importância das corretas medidas epidemiológicas na identificação, isolamento e inativação dos contaminados. O tamanho amostral bem como a ausência de terapia adjuvante controle podem ser apontados como fatores limitantes desta série de casos. Entretanto, a rápida eliminação do vírus em nasofaringe e orofaringe em indivíduos que fizeram uso de protocolo de gargarejo/bochecho com ABF é um resultado promissor no controle da disseminação do SARS-CoV-2, e deve ser objeto de futuras pesquisas.
